# The PharmaResearch Under Eyelid Fine Line Severity Scale: Development and Validation of a Standardized Clinical Assessment Tool for Infraorbital Fine Wrinkles

**DOI:** 10.1111/jocd.70583

**Published:** 2026-01-08

**Authors:** Sun Young Choi, Beom Joon Kim

**Affiliations:** ^1^ Department of Dermatology Chung‐Ang University Gwangmyeong Hospital, Chung‐Ang University College of Medicine Gwangmyeong‐si Gyeonggi‐do Republic of Korea; ^2^ Department of Dermatology Chung‐Ang University Hospital, Chung‐Ang University College of Medicine Seoul Republic of Korea

**Keywords:** grade, under eyelid, validation, wrinkles

## Abstract

**Background:**

Fine lines under the lower eyelid are among the earliest and most prominent signs of facial aging. However, despite growing interest in noninvasive aesthetic procedures targeting this area, there is currently no validated clinical scale specifically designed to assess the severity of infraorbital fine lines. Furthermore, existing assessment tools—such as those for lateral canthal lines or infraorbital hollowing—fail to adequately capture the unique characteristics of horizontally oriented fine wrinkles in this region.

**Aims:**

To develop and validate a novel photonumeric clinical grading tool, called the PharmaResearch Under Eyelid Fine Line Severity (PULS) scale, for the standardized assessment of infraorbital fine lines.

**Methods:**

A total of 53 healthy adults without facial dermatologic conditions were photographed under standardized conditions. From the resulting image set, 73 photographs (53 unique and 20 duplicate images) were selected. Five board‐certified dermatologists used our newly developed 5‐grade photonumeric scale to independently evaluate the severity of infraorbital fine lines. Inter‐ and intra‐rater reliability was assessed using Fleiss' and Cohen's Kappa, respectively. Agreement rates were also calculated.

**Results:**

Inter‐rater reliability analysis yielded a Fleiss' Kappa of 0.6114, indicating good inter‐rater agreement. All raters showed perfect intra‐rater reliability, with Cohen's Kappa values of 1.0000. The overall agreement rate across all evaluations exceeded 80%, supporting the reproducibility and consistency of the PULS scale.

**Conclusions:**

The PULS scale is a validated, reliable, and practical photonumeric grading tool that can address an unmet need in aesthetic dermatology by facilitating standardized evaluation of treatment outcomes for infraorbital fine lines in both clinical research and routine practice.

## 1 Introduction

The infraorbital region, one of the most anatomically delicate and aesthetically significant areas of the face, is often the first to exhibit early signs of aging, including fine lines, laxity, and volume loss [1, 2]. In particular, fine lines under the lower eyelid, which are distinct from crow's feet or infraorbital hollowing, represent a common concern across various age groups; nonetheless, they have received relatively limited attention in clinical research.

Several photonumeric scales have been developed and validated for the evaluation of facial aging features. For example, the Investigator's Global Assessment of Lateral Canthal Line (IGA‐LCL) severity scale [3] and the Allergan Infraorbital Hollows Scale [4] can be used to assess dynamic crow's feet wrinkles and tear trough deformities, respectively. However, no validated clinician‐reported scale currently exists to evaluate infraorbital fine, static, horizontally oriented wrinkles, which exhibit distinct pathophysiological characteristics and therapeutic responses.

The under‐eye area presents unique anatomical and physiological challenges for assessment owing to thin skin, irritation susceptibility, and subtle changes in wrinkle depth. These factors necessitate a highly sensitive, standardized, and reliable assessment tool that can detect small but meaningful clinical changes. Regulatory bodies such as the US Food and Drug Administration emphasize the importance of using validated outcome measures in aesthetic trials [5]. To be accepted as a clinical endpoint, a scale must demonstrate content validity, inter‐ and intra‐rater reliability, and clinically meaningful score changes [5, 6].

In this study, we aimed to introduce and validate a novel photonumeric scale, namely the PharmaResearch Under Eyelid Fine Line Severity (PULS) scale, designed specifically to measure the severity of fine lines below the lower eyelid. This tool was intended to support both clinical trials and real‐world aesthetic practice by offering a validated, reproducible method for the assessment of treatment outcomes in the infraorbital region.

## 2 Methods

### 2.1 Study Design and Participants

This reliability study was conducted to validate the newly developed PULS scale. A total of 53 healthy adult volunteers without any facial skin disease were recruited for standardized digital image acquisition. All participants provided written informed consent after receiving a thorough explanation of the study protocol. The demographic characteristics of the participants whose photographs were included in the scale development are summarized in Table 1.

**TABLE 1:** Participant demographics.

Characteristic	Total (*N* = 53)
Age group, *n* (%)	
≤ 30	8 (15.1%)
30–39	2 (3.8%)
40–49	21 (39.6%)
50–60	22 (41.5%)
Sex, *n* (%)	
Male	0 (0%)
Female	53 (100%)
Ethnicity, *n* (%)	
Asians	53 (100%)

### 2.2 Standardized Photography Protocol

Facial photographs were taken using a Canon EOS 850D camera (Japan) under consistent lighting and environmental conditions, with the camera parameters set as follows: file size, 6000 × 4000 pixels; shutter speed, 1/60 s; aperture, f/8; and ISO, 800. All photographs were taken by the same trained photographer, with the subjects seated upright, looking straight ahead, and their hair pulled back to fully expose the forehead and under‐eye area. The horizontal grid line of the camera was aligned with the under‐eye line, and the vertical grid with the nasal tip, to ensure consistent framing. Each subject's under‐eyelid region was captured in the frontal view. From the final pool of photographs, 73 images (including 53 unique and 20 duplicate images) were randomly selected for scale evaluation.

### 2.3 Scale Development

**FIGURE 1:**
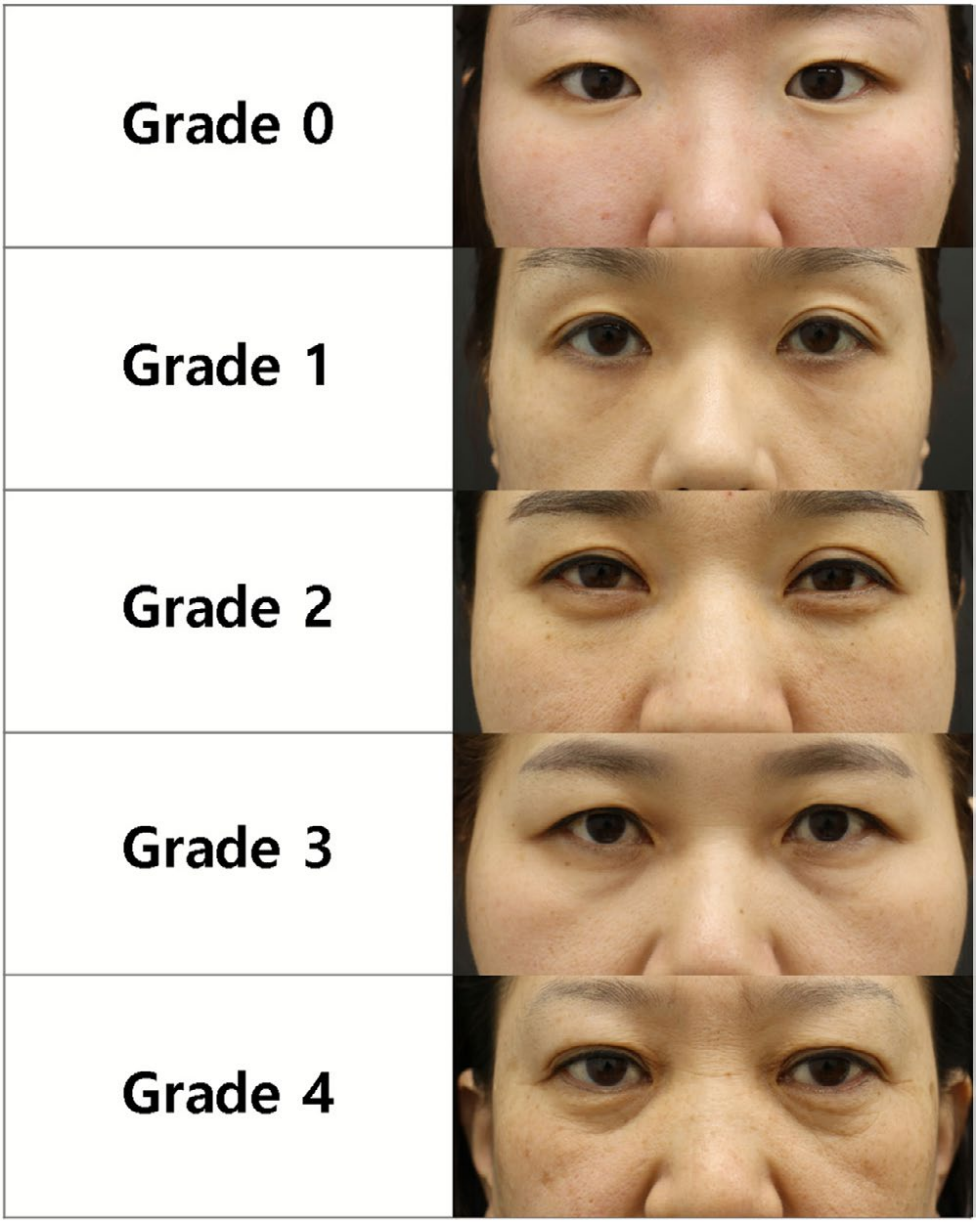
Photo guide of the PULS scale. PULS, PharmaResearch Under Eyelid Fine Line Severity.

The 5‐grade photonumeric PULS scale was developed to assess the severity of fine lines in the under‐eyelid area, with each grade defined as follows: Grade 0 (none), no visible wrinkles or volume loss medially or laterally; Grade 1 (minimal), fine wrinkles with slight volume loss medial to the midpupillary line and a smooth lateral lid‐cheek transition; Grade 2 (moderate), wrinkles extending laterally beyond the midpupillary line with moderate volume loss; Grade 3 (severe), well‐defined wrinkles and a groove along the lid‐cheek junction with moderate volume loss; and Grade 4 (extreme), prominent wrinkles and severe volume loss extending from the medial to the lateral canthus. Representative clinical photographs (Figure 1) and written descriptors (Table 2) for Grades 0–4 are provided to ensure consistent and reproducible assessments. Notably, fine lines were considered to be present beginning at Grade 1, although they were subtle and limited medially at this stage. Their prominence and lateral extension increased progressively in Grades 2 and 3, while Grade 0 explicitly indicated the absence of fine lines.

**TABLE 2:** Grade definitions for the PULS scale.

Grade	Description
0 (None)	No visible wrinkles or volume loss medially or laterally
1 (Minimal)	Presence of wrinkles with some volume loss medial to the midpupillary line; smooth lateral lid–cheek transition
2 (Moderate)	Defined wrinkles extending laterally beyond the midpupillary line with moderate volume loss; smooth lateral lid–cheek transition with mild volume loss
3 (Severe)	Defined wrinkles extending laterally beyond the midpupillary line with moderate volume loss creating a defined groove along the lid–cheek junction
4 (Extreme)	Defined wrinkles extends from medial to lateral canthus; severe volume loss creates a marked step along the lid–cheek junction

Abbreviation: PULS, PharmaResearch Under Eyelid Fine Line Severity.

### 2.4 Rating Process and Raters

Five independent board‐certified dermatologists used the PULS scale to assess the severity of under‐eyelid fine lines. Prior to the assessment process, all raters were trained using comprehensive grading instructions and representative example photographs. The selected images (*n* = 73) were anonymized, randomly renamed, and presented in a randomized sequence. Each rater then conducted separate assessments of the left and right under‐eyelid areas under standardized conditions. To evaluate intra‐rater reliability, we incorporated 20 duplicate images in the assessment series without the raters' knowledge.

### 2.5 Statistical Analysis

All statistical analyses were performed using SAS software version 9.4 or higher (SAS Institute Inc., Cary, NC, USA). Inter‐rater reliability was assessed using Fleiss' Kappa to measure the level of agreement among the five raters [7]. Intra‐rater reliability was evaluated using Cohen's Kappa to determine the consistency of individual raters across repeated images [8]. Kappa values were interpreted according to Altman's classification [9] (Table 3). Unless otherwise specified, all tests were 2‐sided with a significance level of 5%. These statistical approaches align with those previously used to validate existing aesthetic assessment scales for the periorbital region, including photonumeric tools for infraorbital hollows and lacrimal groove wrinkles [4, 10].

**TABLE 3:** Altman's criteria for agreement strength.

Kappa value	Strength of agreement
< 0.20	Poor
0.21–0.40	Fair
0.41–0.60	Moderate
0.61–0.80	Good
0.81–1.00	Very Good

## 3 Results

### 3.1 Inter‐Rater Reliability

The inter‐rater reliability analysis of the PULS scale yielded a Fleiss' Kappa value of 0.6114, indicating good agreement among the 5 dermatologists. When analyzed by laterality (left vs. right), the Kappa value was found to be 0.6155 for the left infraorbital region and 0.6069 for the right infraorbital region. All Kappa values exceeded the threshold of 0.6, confirming acceptable inter‐rater reliability according to Altman's proposed interpretation of the Kappa coefficient [9] (Table 4).

**TABLE 4:** Inter‐rater reliability results.

Category	Fleiss' Kappa	Standard error	95% confidence interval	
			Lower bound	Upper bound
Overall	0.6114	0.021	0.5703	0.6526
Left	0.6155	0.0296	0.5576	0.6734
Right	0.6069	0.0299	0.5482	0.6655

### 3.2 Intra‐Rater Reliability

Intra‐rater reliability was perfect across all the five raters, with each achieving a Cohen's Kappa of 1.0000 for both the left and the right infraorbital region. These results indicate very good agreement within each rater's repeated assessments [8] (Table 5).

**TABLE 5:** Intra‐rater reliability results.

Category	Raters	Cohen's Kappa	Standard error	95% confidence interval	
				Lower bound	Upper bound
Overall	Rater 1	1.0000	0.0000	1.0000	1.0000
	Rater 2	1.0000	0.0000	1.0000	1.0000
	Rater 3	1.0000	0.0000	1.0000	1.0000
	Rater 4	1.0000	0.0000	1.0000	1.0000
	Rater 5	1.0000	0.0000	1.0000	1.0000
Left	Rater 1	1.0000	0.0000	1.0000	1.0000
	Rater 2	1.0000	0.0000	1.0000	1.0000
	Rater 3	1.0000	0.0000	1.0000	1.0000
	Rater 4	1.0000	0.0000	1.0000	1.0000
	Rater 5	1.0000	0.0000	1.0000	1.0000
Right	Rater 1	1.0000	0.0000	1.0000	1.0000
	Rater 2	1.0000	0.0000	1.0000	1.0000
	Rater 3	1.0000	0.0000	1.0000	1.0000
	Rater 4	1.0000	0.0000	1.0000	1.0000
	Rater 5	1.0000	0.0000	1.0000	1.0000

### 3.3 Overall Concordance and Agreement

The percentage agreement for each grade across the raters was above 80%, and the distribution of the assigned grades was consistent with the expected photographic severity. These findings confirm that the PULS scale allows for reproducible and clinically meaningful assessments of under‐eyelid fine lines.

## 4 Discussion

The results of this study demonstrated that the PULS scale is both reliable and reproducible, with good inter‐rater agreement (Fleiss' *K* = 0.6114) and perfect intra‐rater consistency (Cohen's *K* = 1.0000). These findings support its clinical utility as a standardized tool for evaluating under‐eyelid fine lines in both research and clinical settings.

While tools such as the IGA‐LCL severity scale [3] and the Allergan Infraorbital Hollows Scale [4] have been widely used, they do not address the unique presentation of horizontally oriented fine lines below the lower eyelid. In the context of infraorbital aging, it is important to distinguish fine lines (wrinkles, or medically, rhytids) from anatomic creases or folds. Creases and folds represent physiologic or structural skin lines, such as the palpebral crease, and may be present independent of aging. By contrast, rhytids are age‐related changes characterized by dermal collagen and elastin degradation, leading to persistent fine lines. The PULS scale was designed specifically to evaluate rhytids (fine lines) rather than structural folds.

Therefore, the PULS scale was developed to address this gap by offering photonumeric references for subtle changes in infraorbital skin texture and volume. The high inter‐rater agreement observed in this study (> 80%) supports the scale's feasibility and interpretability, and the perfect intra‐rater agreement reinforces its consistency in longitudinal assessments [7, 8, 9].

As recent advances in minimally invasive procedures (e.g., microneedling, laser resurfacing, botulinum toxin injections, and dermal fillers) continue to target the under‐eye region, the need for standardized evaluation tools becomes increasingly critical [11, 12, 13, 14]. Although wrinkling and volume loss are distinct features of aging, they frequently coexist in the infraorbital region. In the PULS scale, wrinkle severity remains the primary target, whereas volume descriptors were included as supportive anchors to facilitate consistent grading and to improve clinical usability. In the absence of such validated scales, cross‐study comparisons remain limited. For instance, Jang et al. demonstrated improvements with magnesium microneedle patches but relied on customized assessment tools [11]. Other technologies such as stereophotogrammetry offer precise but less accessible alternatives [13].

As highlighted by Donofrio et al., standardized grading scales are essential for clinical communication, research validity, and regulatory compliance [14]. The PULS scale offers a balanced, practical solution because it aligns with these principles and contributes to the growing infrastructure needed to support under‐eye aesthetic research.

This study had some limitations. The participant sample was relatively homogeneous and consisted exclusively of Asian individuals, primarily with Fitzpatrick skin types III and IV. While the fundamental biological mechanisms of fine line formation are universal, racial and ethnic differences in skin structure and photoaging patterns may influence the clinical presentation of infraorbital wrinkles. Therefore, the generalizability of the PULS scale to other populations remains to be validated. In addition, assessments were conducted exclusively by board‐certified dermatologists. Thus, future validation studies should include more diverse populations and a broader range of evaluators. Furthermore, longitudinal studies are needed to confirm the scale's sensitivity to treatment‐related changes over time.

In conclusion, the PULS scale is a validated, reliable, and practical photonumeric grading tool that addresses a previously unmet need in aesthetic dermatology. It enables consistent, standardized assessment of under‐eyelid fine lines and is poised to support both clinical research and real‐world practice.

## Funding

This work was funded by PharmaResearch Co. Ltd., Republic of Korea.

## Ethics Statement

The study was approved by the institutional review board (IRB No. P2403‐5909). All participants consented to the reproduction and distribution of any images collected during the study.

## Consent

Written informed consent was obtained from all individual participants included in the study.

## Conflicts of Interest

The authors declare no conflicts of interest.

## Data Availability

The data supporting the findings of this study are available from the corresponding author, KBJ, upon reasonable request.
